# Effect of lairage time prior to slaughter on stress in pigs: a path analysis

**DOI:** 10.1186/s40813-023-00350-w

**Published:** 2023-12-13

**Authors:** Jeongeun Lee, Darae Kang, Kwanseob Shim

**Affiliations:** 1 Department of Agricultural Convergence Technology, Jeonbuk National University, Jeonju, 54896 Korea; 2 Department of Animal Biotechnology, Jeonbuk National University, Jeonju, 54896 Korea

**Keywords:** Pig, Stress, Animal welfare, Hormones, Lairage

## Abstract

**Background:**

Pre-slaughter process during transportation, handling, and lairage causes stress in pigs, affecting animal welfare and meat quality. Therefore, lairage factors are important for relieving stress. A total of 24 LYD (Landrace × Yorkshire × Duroc) barrows were used to investigate the effect of 6 and 20 h lairage time (LT) on cortisol, serotonin, and catecholamine in blood and physiological factors in muscle, and to verify the causal relationship between these factors.

**Results:**

The results revealed that cortisol was increased (0.064 ± 0.007 µg/ml), and epinephrine (0.020 ± 0.002 µg/ml) and norepinephrine (1.518 ± 0.071 µg/ml) were lower at a LT of 20 h than those at 6 h, and there was no significant effect on the muscle and carcass characteristic factors. In addition, cortisol and norepinephrine showed a negative correlation (*r* = -50,346, *p* = 0.0121), epinephrine and glycogen had a positive correlation (*r* = 0.4417, *p* = 0.0307), and serotonin and heat shock protein 70 (HSP70) were positively correlated (*r* = 0.4715, *p* = 0.0200). Path analysis indicated that the increase in LT had a direct effect on cortisol, epinephrine, and norepinephrine, and an indirect effect on muscle glycogen.

**Conclusion:**

This study confirmed the effect of the increase in LT from 6 to 20 h in the lairage room on the stress response of pigs. These findings support the legal requirements that advocate for shorter lairage times, in alignment with enhanced animal welfare standards.

**Supplementary Information:**

The online version contains supplementary material available at 10.1186/s40813-023-00350-w.

## Background

The pre-slaughter processes (handling, loading, transport, and feed deprivation) cause stress in livestock [1–3]. Lairage is a common commercial practice that alleviates stress from pre-slaughter processes and serves as a reservoir on the slaughterhouse’s own schedules [4]. However, lairage can negatively affect animal welfare by mixing with unfamiliar animals, excessively long or short lairage time (LT), fasting and animal handling [4, 5]. However, pigs in the lairage room spend varied times depending on the meat production schedule. Pigs are physically and physiologically affected by LT [6]. In particular, a LT of under 3 h is associated with a higher occurrence of pale, soft, exudative (PSE) meat compared to a LT exceeding 14 h [6–8]. Furthermore, overnight LT leads to a reduction of hot carcass weight and backfat thickness and increases the frequency of firm, dry (DFD) meat, skin damage, gastric ulcers and cortisol levels [7–9]. Thus, proper period of rest in the lairage is an important factor for animal welfare and improving meat quality [6].

Serotonin and dopamine are chemical neurotransmitters in the autonomous nervous system. These hormones are evoked by stress, fear, and reward related responses, which are associated with activation of the hypothalamus-pituitary-adrenal (HPA) axis in response to stress, triggering at self-protecting response [10]. In addition, catecholamines (epinephrine and norepinephrine) are activated by sympathetic-adrenal medullary (SAM) and cortisol are activated by the HPA axis in response to stress, triggering at self-protecting response [11]. Therefore, the psychological and physiological stresses experienced by pigs can be assessed using the state of the HPA axis [12].

A stress reaction is the process of maintaining homeostasis in response to external and internal stimuli in the brain-body response [13–15]. Cortisol and catecholamine affect the dynamic equilibrium in the body during stressful situations [16]. These hormones convert glycogen in muscle into energy and produce lactic acid, which rapidly decreases the pH of the meat and contributes to the formation of PSE meat [16, 17]. Heat shock protein 70 (HSP70) maintains intracellular protein homeostasis as an endogenous protective protein in response to stress [18]. Cortisol [18, 19] and norepinephrine [20] increase HSP70 by influencing stress responses. Thus, hormones and intramuscular factors are closely related. As pigs are vulnerable to stress, it is necessary to appraise stress markers in combination for an accurate evaluation of animal welfare [7]. Therefore, a study that confirms the relationship between hormonal changes and intramuscular physiological factors in the lairage may help improve animal welfare and meat quality.

The interaction of physiological factors due to stress is intricate, and there are few studies that statistically verify how the physiological reaction is caused by the increase in the LT. The animal slaughter detailed regulations of the Animal and Plant Quarantine Agency of Korea stipulate that animal should be rested for appropriate amount of time not to exceed 12 h. In Korea, the average lairage is known to be 5–6 h [21]. Accordingly, in this study, the effects of excessive retention time on stress-related hormones in the blood and intramuscular factors were evaluated. We hypothesized that hormonal pathways are triggered by lairage. We explored the causality between individual items and the indirect effect by LT through the path analysis.

## Results

### Effects of LT on hormones, body weight, carcass weight, backfat thickness, and muscle related parameters in pigs

Hormone levels in the serum, body weight, carcass weight, backfat thickness, glycogen, and muscle related parameters were investigated to evaluate the psychological and physiological stress caused by the period of lairage (Table 1). There were no differences in dopamine and serotonin levels between the 6 and 20 h groups. Cortisol was affected by LT (*p* < 0.01) and was higher in the 20 h lairage group. However, the epinephrine and norepinephrine levels were significantly lower in the 20 h lairage group (*p* < 0.01). Moreover, LT had no effect on body weight, carcass weight, backfat thickness, glycogen, lactic acid, and HSP70. HSP70 protein bands are shown in Fig. S1.

**Table 1:** Effects of 6 and 20 h lairage time (LT) on hormone in serum, body weight, carcass weight, backfat thickness, and muscle parameters in pigs

Items	LT		P-value
	6 h	20 h	
Dopamine (µg/ml)	0.061 ± 0.003	0.052 ± 0.003	0.0993
Serotonin (µg/ml)	0.547 ± 0.065	0.683 ± 0.084	0.2127
Cortisol (µg/ml)	0.041 ± 0.005	0.064 ± 0.007^**^	0.0099
Epinephrine (µg/ml)	0.040 ± 0.004^**^	0.020 ± 0.002	0.0007
Norepinephrine (µg/ml)	1.884 ± 0.037^**^	1.518 ± 0.071	0.0003
Body weight (Kg)	117.850 ± 1.747	117.525 ± 1.912	0.9013
Carcass weight (Kg)	90.167 ± 1.336	87.917 ± 1.427	0.2622
Backfat thickness (mm)	21.333 ± 0.964	20.500 ± 0.839	0.5212
Glycogen (µg/ml)	1.191 ± 0.087	1.073 ± 0.056	0.2683
Lactic acid (µg/ml)	3.421 ± 0.072	3.396 ± 0.030	0.7550
HSP70 protein	1.544 ± 0.082	1.562 ± 0.115	0.8970

Values are presented as mean ± SE, n = 12, **p < 0.01, significantly different between 6 and 20 h groups, HSP70 was normalized with GAPDH. LT: Lairage time, SE: Standard Error, HSP70: Heat shock protein 70, GAPDH: Glyceraldehyde 3-phosphate dehydrogenase

### Relationship between hormones and muscle related parameters

Pearson correlation analysis was performed to investigate the relationship between the variables (Table 2).

Serotonin concentrations were positively correlated with HSP70 levels (*p* < 0.05). Cortisol and Norepinephrine concentrations also showed negative correlations with each other (*p* < 0.05). Among, there was a significant positive association between glycogen and epinephrine (*p* < 0.05). In addition, HSP70 and backfat thickness were positively correlated (*p* < 0.05), and carcass weight and body weight were significantly positive associated (*p* < 0.01). However, dopamine showed a statistical tendency to be positively correlated with epinephrine (*p* = 0.0614), norepinephrine (*p* = 0.0666), and glycogen (*p* = 0.0869). In addition, there was a positive correlation tendency between glycogen and lactic acid (*p* = 0.0977).

**Table 2:** Pearson correlations between stress related parameters and carcass characteristics in pigs

Parameters	Dopamine	Serotonin	Cortisol	Epinephrine	Norepinephrine	Glycogen	Lactic acid	HSP70	Body weight	Carcass weight	Backfat thickness
Dopamine	1	0.17441	-0.12525	0.38742^†^	0.38046^†^	0.35687^†^	-0.0782	0.17313	-0.24979	-0.16744	-0.31124
		(0.4150)	(0.5598)	(0.0614)	(0.0666)	(0.0869)	(0.7164)	(0.4185)	(0.2391)	(0.4342)	(0.1388)
Serotonin		1	0.15296	-0.23372	-0.33613	0.1021	0.11875	0.47151^*^	-0.15803	-0.21289	0.23589
			(0.4755)	(0.2717)	(0.1083)	(0.6350)	(0.5805)	(0.0200)	(0.4608)	(0.3179)	(0.2671)
Cortisol			1	-0.01153	-0.50346^*^	0.23945	0.1128	-0.16196	-0.10075	-0.20653	-0.20126
				(0.9574)	(0.0121)	(0.2598)	(0.5997)	(0.4496)	(0.6395)	(0.3329)	(0.3457)
Epinephrine				1	0.37565^†^	0.44173^*^	0.04868	-0.26513	0.12583	0.26485	-0.10158
					(0.0705)	(0.0307)	(0.8213)	(0.2105)	(0.5580)	(0.2110)	(0.6367)
Norepinephrine					1	0.24749	-0.1642	0.10269	-0.09706	0.05616	0.03047
						(0.2436)	(0.4433)	(0.6330)	(0.6519)	(0.7944)	(0.8876)
Glycogen						1	0.34603^†^	0.2142	-0.11627	-0.06344	-0.05825
							(0.0977)	(0.3149)	(0.5885)	(0.7684)	(0.7869)
Lactic acid							1	0.18238	-0.0603	-0.04774	0.08369
								(0.3937)	(0.7795)	(0.8247)	(0.6974)
HSP70								1	-0.10532	-0.10795	0.40779^*^
									(0.6243)	(0.6156)	(0.0479)
Body weight									1	0.97714^**^	0.22967
										(< 0.0001)	(0.2803)
Carcass weight										1	0.24811
											(0.2424)
Backfat thickness											1

The numbers in parentheses mean P-values. Correlation significant at †*p* < 0.1, *p < 0.05, **p < 0.01. HSP70: Heat shock protein 70

### Path analysis

**Fig. 1:**
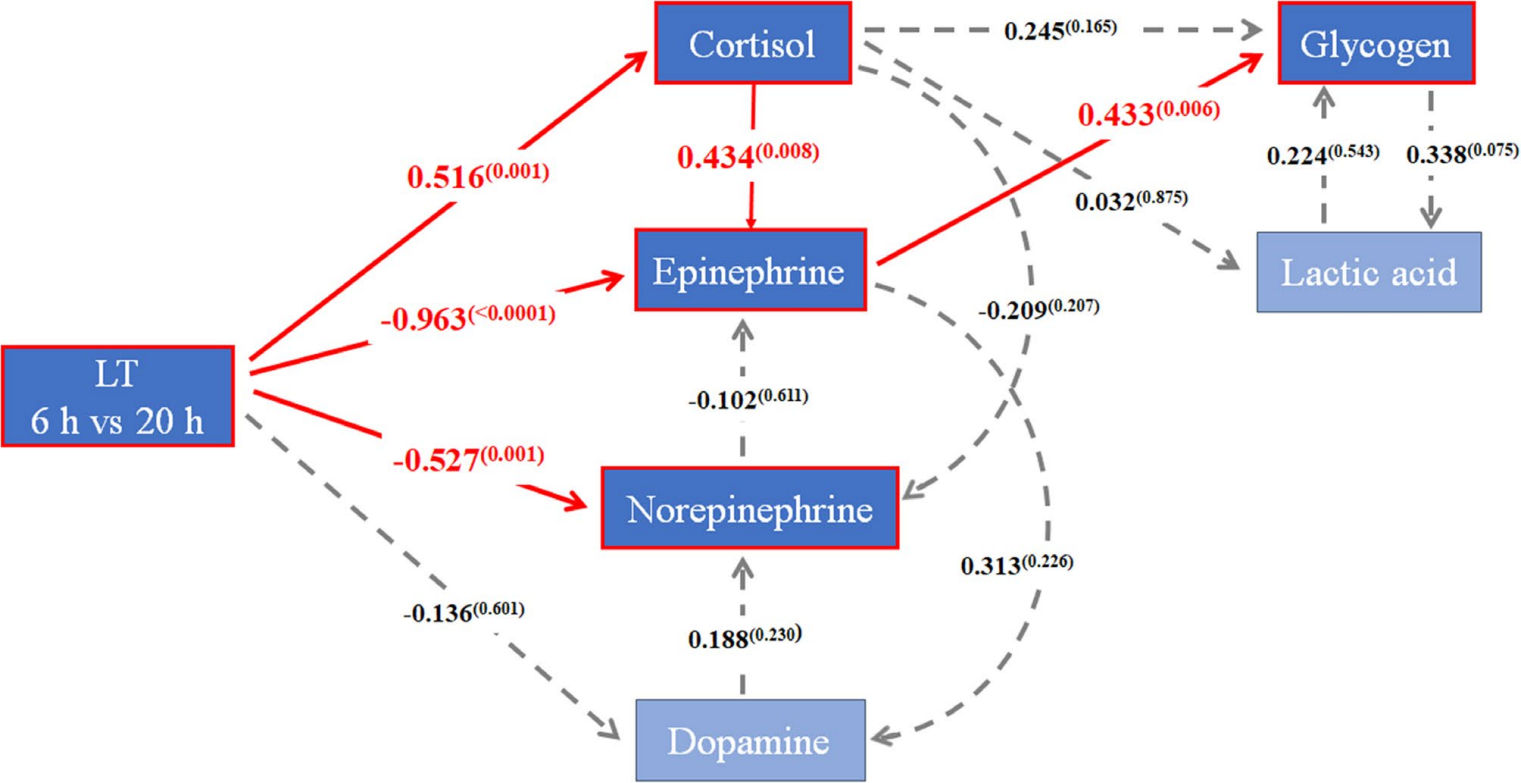
Path analysis of association among hormones in blood, glycogen, and lactic acid according to increase in lairage time in the slaughterhouse. Values on the arrows represented the standardized coefficients and the superscripts indicate the p-value. Bold red line arrows indicate significant associations and the gray dotted arrows indicate the non-significant associations. n = 24. LT: lairage time 6 h vs. 20 h

Based on the results of the correlation analysis, we verified the hypothesis using the model’s goodness-of-fit (Pr > Chi-square = 0.300, GFI = 0.920, CFI = 0.971). Figure 1 shows a diagram of the path analysis results with standardized estimates of the associations among the cortisol, epinephrine, norepinephrine, dopamine, glycogen, and lactic acid. The hypothesis model that included serotonin and HSP70 did not meet the model’s goodness-of-fit. As a result, HSP70 and serotonin were not included in the path analysis results. One arrow starting from the LT 6 h vs. 20 h indicating an increase in holding time represents a direct effect, and two arrows from the LT 6 h vs. 20 h to the factors indicate an indirect effect. First, the LT affects hormones in the blood. Increasing LT was directly positively associated with cortisol (0.516, *p* < 0.01) and had a directly negative effect on epinephrine(-0.963, *p* < 0.01) and norepinephrine (-0.527, *p* < 0.01). However, when considering an indirect pathway from cortisol to epinephrine, epinephrine exhibited an indirectly positive effect (0.434, *p* < 0.01) due to increasing LT. Increasing LT was indirectly positive associated with glycogen (0.433, *p* < 0.01). The indirect effect of glycogen was manifest through two distinct pathways: one involved the impact on glycogen via cortisol and epinephrine, while the other pathway depicts epinephrine affecting glycogen. Additionally, there was an observed tendency of glycogen to positively affect lactic acid levels (0.338, *p* = 0.075). However, increasing the time in slaughterhouse from 6 to 20 h had no significant direct or indirect effect on dopamine.

## Discussion

Pigs are stressed by the series of processes they undergo during transport and before slaughter [1–3]. Elevated stress levels during lairage have the potential to adversely affect meat quality, a phenomenon that becomes particularly pronounced in relation to the duration of stay within the lairage facility [6]. Therefore, establishing appropriate LT to relieve stress is important to improve animal welfare and meat quality. In previous studies, although the effect of meat quality and carcass quality as well as physiological parameters were examined according to LT in slaughterhouses [6–8], no study has statistically examined the interrelationship on how hormones responding to stress affect physiological factors in the muscles. In this study, we investigated the causal relationship between each factor based on 6 h vs. 20 h LT.

Cortisol is a glucocorticoid synthesized from cholesterol, and secreted by the adrenal cortex [22]. Cortisol, as a representative biochemical marker of stress [23], has been extensively investigated in studies to evaluate stress induced by lairage. In a previous study, rest in the lairage room for 24 h increased cortisol concentration and skin blemish scores [7]. Zhen et al. (2013) [24] reported that the pigs slaughtered immediately after transport exhibited higher levels of cortisol compared to those slaughtered after a 3 h period of lairage. However, there were no differences in cortisol levels between the 2 and 24 h lairage groups [25]. Nevertheless, most previous studies have described that a long LT is more stressful for livestock. Table 3 summarizes the results of previous studies that observed changes in cortisol related to LT. In this study, the concentration of cortisol was higher in the 20 h lairage group than in the 6 h lairage group. This result was consistent with previous studies which demonstrated an elevation in blood cortisol concentration with an increase in LT [7, 24]. In addition, the path analysis showed that the increase in LT from 6 to 20 h had a positive and direct effect on cortisol, signifying that an increase in retention time from 6 to 20 h in the lairage stage directly affected the increase in cortisol.

**Table 3:** Overview of previous studies on the effect of increasing LT on cortisol

Reference	LT	Cortisol	Additional information
Zhen et al., 2013 [24]	0 h, 3 h, 8 h, 24 h	↑	more stressful
Dokmanović et al., 2014 [6]	8 min-2.7 h, 14-21.5 h	↔	more stressful
Dokmanović et al., 2017 [7]	1 h, 24 h	↑	more stressful
Salajpal et al., 2005 [25]	2 h, 24 h	↔	-
Gispert et al., 2000 [41]	< 3 h, 3–9 h, > 9 h	↑	more stressful

↑: means that cortisol increased as the LT increased, ↔: means that no differences are observed. LT: lairage time

Catecholamines (dopamine, norepinephrine, and epinephrine) originate all from the L-tyrosine. Dopamine is synthesized first among the catecholamines. This process involves the enzymatic conversion of L-tyrosine into dopamine through the interplay of tyrosine hydroxylase, tetrahydrobiopterin and oxygen. Following dopamine is synthesis, dopamine is converted by β-hydroxylase with ascorbate and oxygen to modify it to norepinephrine in the peripheral tissue. Thereafter, norepinephrine is converted into epinephrine through the action of phenylethanolamine N-methyltransferase (PNMT). Through this synthetic step, stressed animals increase the synthesis and utilization of catecholamine (norepinephrine and epinephrine) in the adrenal medulla via activation of the sympathetic nervous system [26, 27]. The adrenal cortex secretes cortisol via signals from the hypothalamus and anterior pituitary in a stressful situation [22]. Cortisol can affect epinephrine levels by regulating the enzyme PNMT [16, 28]. This paracrine action of cortisol was also observed in this study. In the path analysis, we observed that concentration of cortisol increased as the time that pigs spent in lairage increased from 6 to 20 h. We also found that cortisol had a positive effect on epinephrine. However, cortisol had no direct or indirect effect on norepinephrine. In addition, LT has been shown to directly affect levels of epinephrine and norepinephrine. These catecholamines are typically regulated by SAM axis [16]. Nonetheless, this result indicates that stress during lairage triggers the HPA axis to release cortisol, which can regulate PNMT. PNMT, in turn, affects epinephrine levels. Therefore, this study suggests that stress-induced activation of the HPA axis during lairage can modulate epinephrine concentration by regulating PNMT.

Glycogen and lactic acid represent the energy metabolism in the body [24]. The pre-slaughter stress response releases cortisol and catecholamine to maintain body homeostasis [16], and this response increases the use of intramuscular glycogen and the conversion of glycogen to lactic acid [16, 17]. Moreover, in animals experiencing stress, the depletion of glycogen in the muscles result in an elevation of lactic acid levels, leading to the hydrolysis of adenosine triphosphate [29]. During this process, HSP70 levels increase in the muscle tissue to preserve intracellular protein homeostasis [18, 29] This study showed that cortisol had an indirect effect on glycogen, and epinephrine had a direct effect on glycogen as a result of activation of HPA axis according to the increase in LT. In a study of Zhen et al. (2013) [24], intramuscular lactic acid levels were measured at 0 and 3 h of LT and showed a decrease in lactic acid at 3 h group compared to 0 h group. This means that glycolysis was reduced due to LT. Despite this, in the case of long term lairage, glycogen is reduced due to increased fighting and hunger during lairage, which may increase the lactic acid in the muscle [24, 30]. In this study, the lactic acid levels and HSP70 were not affected. This is because the effect of a 20 h rest time in the slaughterhouse may not have reached the stepwise change of glycogen to lactic acid and HSP70 expression. In particular, HSP70 and serotonin exhibited a positive correlation in the correlation analysis. Serotonin, a neurotransmitter, is commonly referred to as 5-hydroxytruptamine (5-HT) [31]. El-Kasaby et al. (2014) [32] showed that HSP70 interacts with the serotonin transporter, which reuptakes serotonin into the presynaptic neuron, when it is in endoplasmic reticulum. We also found that glycogen reduction due to the increase in rest time in the slaughterhouse is actively mediated by the HPA axis rather than the sympathetic nervous system (norepinephrine). Hormone and neurotransmitter activities vary depending on the cause of stress and magnitude of the response [33]. However, since we did not analyze all of the intermediate factors involved in the stress response, further research is needed to explain this effect accurately.

Cortisol and catecholamine hormones change in response to stress [33]. However, as mentioned above, stress responses occur in various states depending on the individual condition and stress. Therefore, interpreting the emotional and physical circumstances is necessary. Psychological stressful situations, such as mixing with unfamiliar individuals or being placed in a crowded situation, can cause physiological changes in pigs [34, 35]. Pigs are known to have elevated cortisol levels and vigorous social responses when crammed with pigs [36–38]. In addition, norepinephrine decreased after 2 h of exposure to social stress (regrouping) compared to immediate social stress exposure [39]. Pigs experience social stress when in a lairage room. In the present study, cortisol increased from 6 to 20 h of LT, whereas norepinephrine and epinephrine levels decreased.

The lairage room functions as a holding area integrated within the slaughterhouse’s operational timetable [4]. Consequently, the duration of pigs’ stay in the lairage room varies in accordance with the meat production schedule. In this study, a duration of 6 h was chosen to align with the average time in Korea and 20 h LT was selected to exemplify a period exceeding the established limit of 12 h. The decision to implement a lairage time exceeding 12 h in this slaughterhouse was exclusively made for our experiments, specifically for the purpose to reinforce the legal requirement. However, the careful interpretation is required since we assumed a scenario where the 6 and 20 h LT aligned with the slaughterhouse schedule, resulting in different slaughter times for the two groups. Although both groups were slaughtered on the same day, they underwent slaughter at different times. The 6 h LT group was transported from the farm in the early morning and slaughtered in the afternoon, while the 20 h LT group was slaughtered in the morning after being transported the previous afternoon. Furthermore, while the transfer time, travel route, and type of truck remained consistent, it is important to note that there could have been differences in the drivers involved.

## Conclusion

This study contributes to a better understanding of how stress response in pigs is caused by changing the LT from 6 to 20 h. We have shown that it is possible to investigate the pathway in which complex physiological reactions occur with increasing LT. Our investigations reveal that prolonged LT induce heightened stress, impacting intramuscular factors either directly or indirectly through stress-related hormones specifically cortisol and epinephrine. Furthermore, our observations suggest that the stress induced by a 20 h LT triggers the activation of the HPA axis, potentially influencing epinephrine concentration by regulating PNMT. Hence, this approach not only enriches our insight into the intricate workings of stress hormone mechanisms but also lends support to the notion that adhering to shorter lairage times (6 h) aligns with enhanced animal welfare requirements.

## Methods

### Animals and experimental design

The study was carried out in the Republic of Korea, during the summer (July, 2021). All pigs were raised on the same commercially certified animal welfare farm adhering to stringent animal welfare standards to mitigate additional stressor and fed a standard commercial diet. A total of 24 LYD (Landrace × Yorkshire × Duroc) barrows with a mean body weight of 129.7 ± 12 kg were included in study. All pigs were slaughtered in the commercial slaughterhouse in Anseong (Korea) which is certified as an animal welfare slaughterhouse by the Ministry of Agriculture, Food and Rural Affairs, Republic of Korea. This choice was made to reduce stress on animals within the slaughterhouse environment. The pigs were randomly divided into two treatment groups on the farm for different LT. The 6 h LT was transported from the farm to the slaughterhouse in early morning on the day of slaughter, and the 20 h LT was transported in the afternoon of the previous day of slaughter. The pigs were transported with same truck and transportation time was 1 h. The loading ramp was inclined downward at an angle not exceeding 20° and were kept in the same size lairage pen (0.83 m^2^ per pig) for either 6 and 20 h. Pigs transported together were maintained in groups during the LT, they were not mixed with other groups, and afforded unrestricted access to water. The 6 h LT was selected from the most common LT used in standard industrial slaughterhouses and 20 h was chosen as the excess LT. All pigs were slaughtered following the guidelines of the Livestock Sanitation Management Act (Livestock Products Sanitary Control Act; Act No. 18,445, 2021 revision), with approximately 400 pigs processed per hour. All pigs were slaughtered on the same day, but slaughtered at different times. The 6 h LT group was slaughtered in the afternoon and the 20 h LT group was slaughtered in the morning. The other conditions related to slaughter and transport were kept the same to compare the effects of LT. Carcass weight and backfat thickness were measured on a hanging carcass.

### Blood sampling and determination of hormone levels

Blood was collected in 50 ml conical tube during the exsanguination phase of the slaughter process and immediately transported to the laboratory. Samples were kept at 4 ℃ during transport to the laboratory. The collected blood was centrifuged at 2000 × g for 10 min at 4 ℃, and the serum was collected. The serum was stored at -80 ℃ until analysis. Before analysis, serum was diluted in methanol (1:9, v/v) and vortexed. After incubation at -20 ℃ for 1 h, all were centrifuged at 18,000 × g for 10 min at 4 ℃, and the supernatant was used for liquid chromatography with tandem mass spectrometry (LC-MS/MS) installed in the Center for University Wide Research Facilities (CURF) at Jeonbuk National University. Dopamine, serotonin and cortisol were profiled using a Waters Xevo TQ-S (Waters Corporation, Milford, MA, USA), and epinephrine and norepinephrine were determined using an Agilent 6410B (Agilent Technologies, Palo Alto, CA, USA). The injection volumes were 5 µL and 3 µL, respectively. The chromatographic separation was performed on an Synergi Hydro-RP column (4 μm, 2 × 150 mm) with a flow rate of 0.2 mL/min at 45 ℃. Mobile phase A was 0.1% formic acid in distilled water and mobile phase B was 0.1% formic acid in methanol. The mobile phase in gradient program of Waters Xevo TQ-s was set as follows: 0–1 min 0% B, 1–4 min linear increase to 100% B, 4-4.5 min 100% B, 4.5-5 min linear decrease to 0% B, 5–10 0% B. The Agilent 6410B gradient was 0–1 min 0% B, 1–10 min linear increase to 100% B, 10–11 min 100% B, 11–14 min linear decrease to 0% B, and 14–20 min 0% B.

### Muscle sampling and determination of physiology factors

After slaughter, muscle tissue was collected from the longissimus dorsi muscle of pigs. Muscle tissues were frozen in liquid nitrogen and stored at − 80 ℃ until analysis. Muscle glycogen concentration was measured using a commercial glycogen assay kit (Abcam, Cambridge, UK) according to the manufacturer’s instruction.

Lactic acid extraction and quantification in muscle tissue were performed according to the method described by Visessanguan et al. (2004) [40] with minor modifications. Briefly, 3 g of fat-removed muscle tissue were homogenized for 30 s in 27 mL of distilled water and centrifuged at 3000 × g for 10 min. The supernatant (600 µL) was deproteinized with 1.2 mL of 0.5 N perchloric acid (Deajung, Seoul, Korea). After incubation for 5 min at room temperature (RT), the samples were centrifuged at 12,000 × g for 10 min. The supernatant was used for high-performance liquid chromatography (HPLC) analysis after filtering using a 45 μm membrane filter. Lactic acid content was quantified using HPLC equipped with Aminex HPX-87 H column, 7.8 × 300 mm (BioRad, Richmond, CA, USA), and the column temperature was set at 30 ℃. The wavelength of the UV PDA detector was set to 220 nm. The mobile phase in gradient program was set as follows: flow 0.6 mL 0.2% phosphoric acid for 26 min, 70% acetonitrile with 0.2% phosphoric acid (v/v) for 1 min, 0.2% phosphoric acid for 12 min. Lactic acid in the muscles was determined based on the peak time and area of the lactic acid standard (Sigma Aldrich, MO, USA).

HSP70 protein expression was measured using western blotting. Total protein was extracted from 100 mg of muscle tissue using RIPA buffer (Biosesang, Sungnam, Korea). After homogenization, the samples were centrifuged and the supernatants were collected. Protein concentration was determined using DC kit (Bio-Rad, Hercules, CA, USA). Equal amounts of proteins (20 µg) were separated using SDS-PAGE. The proteins that separated by 12% acrylamide gel were then transferred onto polyvinylidene fluoride (PVDF) membranes. After transfer, blocking was performed for 1.5 h with 5% skim milk in Tris buffered saline with Tween 20 (TBST; 2 mM Tris, 13.7 mM sodium chloride, 0.5 mM potassium chloride, 0.05% Tween 20) at RT. The membranes were then rinsed thrice with TBST and incubated with primary antibodies against glyceraldehyde 3-phosphate dehydrogenase (GAPDH, 1:5,000, Invitrogen, Carlsbad, CA, USA) and HSP70 (1:2,500, Enzo, San Diego, CA, USA) with 5% skim milk in TBST overnight. After the primary antibody incubation, the membrane was washed thrice with TBST, and the goat anti-mouse secondary antibodies (1:5,000, Enzo, San Diego, CA, USA) were incubated at RT for 1.5 h. After washing with TBST, the specific protein bands were visualized via an ECL kit (Thermo Fisher, San José, CA, USA) and the image of protein bands were detected by iBright CL100 Imaging system (Thermo Fisher, San José, CA, USA). All protein data were normalized to that of GAPDH.

### Statistical analyses

All data were analyzed using the SAS statistical software (version 9.4; SAS Institute Inc., Cary, NC, USA). One-way ANOVA and t-test were used to compare the effects of LT on hormones, glycogen, lactic acid, HSP70, and carcass characteristics in pigs. Subsequently, Pearson correlation analysis was used to determine the association between hormones in the blood, glycogen, lactic acid, and HSP70. Considering the results of the correlation analysis, we synthesized the effect of increasing LT, and path analysis was performed to investigate the direct and indirect effects of hormones on blood and muscle parameters. Several models were tested to determine the relationships between variables. Model fit was assessed using the Pr > Chi-square, goodness of fit index (GFI), and comparative fit index (CFI). All data are presented as mean ± standard error (SE). Statistical significance was set at *p* < 0.01, *p* < 0.05, and *p* < 0.1.

### Electronic supplementary material

Below is the link to the electronic supplementary material.

**Supplementary Material 1**: Figure S1. The results of the western blot analysis. Heat shock protein 70 (HSP70) protein expression levels normalized by glyceraldehyde-3-phosphate dehydrogenase (GAPDH)

## Data Availability

The data are available from the corresponding author upon reasonable request.
