# Supplementary material for: Effect of lairage time prior to slaughter on stress in pigs: a path analysis
Source: Porcine Health Manag. 2023 Dec 13;9:55. doi: 10.1186/s40813-023-00350-w (PMC10717777; doi:10.1186/s40813-023-00350-w)
Supplement: Supplementary file 1 — Supplementary Material 1: Figure S1. The results of the western blot analysis. Heat shock protein 70 (HSP70) protein expression levels normalized by glyceraldehyde-3-phosphate dehydrogenase (GAPDH) [file 40813_2023_350_MOESM1_ESM.docx]

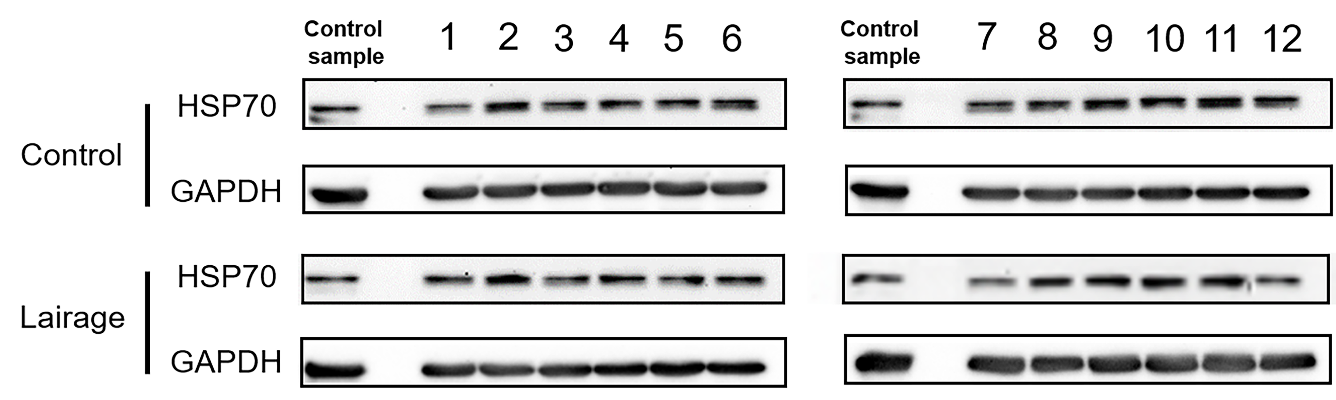


**Supplementary Figure S1** The results of the western blot analysis. Heat shock protein 70 (HSP70) protein expression levels normalized by glyceraldehyde-3-phosphate dehydrogenase (GAPDH).
